# Influence of extracellular protein isolated from fish gut associated bacteria as an enhancer of growth and innate immune system in *Mugil cephalus*

**DOI:** 10.1038/s41598-022-05779-7

**Published:** 2022-02-25

**Authors:** Rajasekar Thirunavukkarasu, Priyadarshin Pandi, Deivasigamani Balaraman, Fadwa Albalawi, Naushad Ahmad, Mani Panagal, Tentu Nageswara Rao, Kumaran Subramanian, Edward Gnana Jothi George, MaryShamya Arockia Rajan, Pugazhvendan Sampath Renuga, Wilson Aruni, Suliman Yousef AlOmar

**Affiliations:** 1 Centre for Drug Discovery and Development, Sathyabama Institute of Science and Technology, Chennai, Tamil Nadu 600 119 India; 2 Centre of Advanced Study (CAS) in Marine Biology, Annamalai University, Parangipettai, Cuddalore, Tamil Nadu 632502 India; 3 Department of Zoology, King Saud University, Riyadh, 11451 Kingdom of Saudi Arabia; 4 Chemistry Department, College of Science, King Saud University, Riyadh, 11451 Kingdom of Saudi Arabia; 5 Department of Biotechnology, Annai College of Arts & Science, Kumbakonam, Tamil Nadu India; 6 Department of Chemistry, Krishna University, Machilipatnam, Andhra Pradesh 521001 India; 7 Kemin Industries South Asia Private Limited, KeminAquaScience™, Ambattur Industrial Estate, Chennai, 600 058 India; 8 Arignar Anna Government Arts College, Cheyyar, Tamil Nadu 604407 India; 9 Musculoskeletal Research Center, The Loma Linda Veteran Affairs, United States Department of Veteran Affairs, Loma Linda, CA 92354 US; 10 Department of Biotechnology, Mohamed Sathak College of Arts & Science, Sholinganallur, Chennai, Tamil Nadu 600119 India; 11 Amity University, Mumbai, Maharashtra India

**Keywords:** Marine biology, Animal biotechnology, Applied immunology

## Abstract

The cultural microbiomes of 27 bacteria colonies were isolated from *Mugil cephalus for* analysis of the antibacterial and antagonistic activities. A potent probiotic bacterium was characterized using16S r RNA sequencing. The potent strain was added to fish diet to perform the challenge test and to study the growth and immunological parameter. The extracellular proteins from the probiotic were collected and characterized using MALDI TOF/TOF. Out of G27, G9 strain inhibited all the five pathogenic strains. An isolated bacterium was identified as *Bacillus subtilis* PRBD09 with accession number KF765648. After 35 days of feeding period *B. subtilis* PRBD09 enhance the both cellular and humoral immune responses, which responsible for survive of the *Mugil cephalus* against *Aeromonas hydrophila* infection*.* The MALDI TOF sample 08 and 09 were recognized as hypothetical proteins based on the MALDI TOF sample. A cytidinedeaminase was found in samples 10, 11, and 12. Extracellular proteins may be involved for the immunological increase in *Mugil cephalus* against *Aeromonas hydrophila*, according to the current research.

## Introduction

Every year, billions of dollars are lost in global aquaculture due to bacterial infection, posing a severe threat to the long-term viability of fish farms^1^. *Aeromonas* septicemia is the most common disease in aquaculture^2^. Recent finding reported routine use of chemical such as antibiotics in the aquatic environment it leads to increasing of antibiotic resistance bacteria^3,4^. Many alternatives methods are used in the aquaculture such as dietary administration of feed additives. Probiotic was most important feed supplement for control or treatment to the disease of fish in the aquaculture^5^. Chemotherapy also disrupts gut homeostasis of gut physiology, and cause fish to be vulnerable to infections. Consequently probiotic was the alternative to disease treatment^6^.

Probiotic serve as a prophylaxis treatment in the microbial infection, its directly inhibiting the pathogen's proliferation^7^. Some researchers have shown that finding an alternative to antibiotics and treatments with fewer adverse effects is possible^8^. The interest of probiotic and changes in intestinal microbes arose primarily to support and maintain and attachment sites, adhere to the gut, and inhibit pathogen growth for nutrients and valid alternative to prophylactic antibiotics and biocides^9^. Fish feed with probiotics which enhance the growth and disease resistant^10^. There are numerous reports available about Probiotic feed for human, ruminant and poultry. While use of Probiotic fish feeds in the aquaculture is new concept^11^.

Probiotics enhance the development of the host as well as the immunological response to various infections. Research has consistently demonstrated that probiotics benefit growth, food metabolism, immune responses, sickness prevention, the gut microbiota, and water quality^12^. Many reports suggested that inhibition of microbial pathogens, and increase the fish yields and enhance the fish immune response^13^. Probiotic are able to suppress the growth of the microorganisms through the various mechanisms such as immune system regulation, antimicrobial chemical entities, antimicrobial compounds and adhesion to epithelial cells^14^.

*LactoBacillus* can release antimicrobial compounds such as bacteriocins, ethanol, fatty acids and hydrogen peroxide. This type of compounds suppress the various type of microorganisms such as *Escherichia coli*^15^, *Shigella *spp.^16^, *Streptococcus mutans*^17^, *Pseudomonas aeruginosa*^18^, and *Staphylococcus aureus*^19^ and including *Clostridium difficile*^20^.

*Bacillus *spp. was used in aquaculture because it produced a hydrolytic enzyme that increases nutrient intake. Gut microbes create hyrdrolytic enzymes, which increase the availability of phosphorus and other nutrients. Hydrolyzing phytate is likely to decrease its anti-nutritional effect. Because fish lack phytase enzymes, they require supplementary inorganic phosphate in their diet to meet their phosphate requirements and guarantee optimum growth^21^. Microbial phytase has been proven to improve phytate phosphorus bioavailability and hence reduce the use of inorganic phosphorus supplements in chicken and fish^22–24^. Aqua-feed businesses have long employed hydrolytic enzymes to improve fish development, nutrient utilisation, and bioavailability of macro and micro minerals, as well as to reduce faecal phosphorus pollution into the aquatic environment^25^. *Bacillus *spp. are frequently used as probiotics because to their potential to create endospores that are resistant to the low pH of the gastrointestinal tract^26^. Directly testing efficiency of potential probiotics on food fish species, on the other hand, sometimes necessitates a large culture area and is expensive.

The grey flathead mullet (*Mugil cephalus* L.), also known as the striped is a coastal fish species found all over the world. *Mugil cephalus* has a bright future in Europe, East Asia, and South Asia. It is a significant aquaculture species in India at the same time. Many studies on the main haematological and biochemical parameters in this fish species under various settings have been published^27^. However, there is a insufficiency of research on the benefits of probiotics on marine fish. There have been very few investigations on the effect of an Immunostimulation probiotic in mullets. Hence, in the present studies were carried out with the potential probiotic actions of *Mugil cephalus* aganist *A. hydrophila* were investigated.

## Materials and methods

### Ethical statement

The experiment was carried out in accordance with the Institutional Ethical Committee (IEC) of Sathyabama Institute of Science and Technology (1793/PO/REBI/S/2014/CPCSEA) Chennai, norms and regulations. The Institutional Animal Ethical Committee (IAEC) of Sathyabama Institute of Science and Technology authorised all animal experimental procedures. Throughout the investigation, the ARRIVE guidelines (https://arriveguidelines.org/arrive-guidelines/experimental-procedures) were followed.

### Sample collection and acclimatization

All of the fish were caught at the Vellar estuary, Protonovo, Tamil Nadu. The fish were acclimated in fibre tanks with UV-treated estuary water with salinities ranging from 28 to 30 ppt, temperatures ranging from 22 to 28°C, and commercial fish pellets (Probfloc aqua probiotics) (4% body weight). A bio filter was utilised to aerate and recycle the water in the tank on a constant basis. On a daily basis, fresh aerated estuary water replaced 15% of the overall water volume.

### Probiotic bacteria isolated from fish

Fish (*M. cephalus*) was used to isolate the probiotic bacterium. In the lab acclimatised the live fish. After disinfecting the ventral surfaces with 70% ethanol, the intestine was removed aseptically from the fish abdominal cavity. It was meticulously treated with sterilized cold normal saline solution to remove feed materials, grime, and other impurities (NSS). Before the homogenization fish intestine were weighted and macerating it with a sterile glass rod. During the homogenizing use the sterile NSS (1:10 w/v) with a vortex mixer to eliminate excess moisture. To measure the total plate count, samples of the fully macerated and homogenised intestine were serially diluted in NSS and aseptically plated onto nutritional agar using the spread plate technique. The total bacteria were counted after the inoculation agar plates had been incubated at 22–24 °C for 24–48 h.

### Test pathogens

The Microbial Type Culture Collection (MTCC), Chandigarh, India, provided pathogenic bacterial strains such as *Aeromonas hydrophila* (MTCC 646), *Vibrio alginolyticus* (MTCC 4182), *Vibrio harveyi* (MTCC 3438), *Vibrio parahaemolyticus* (MTCC 451), *Vibrio vulnificus* (MTCC1145).

### Antibacterial properties of the probiotic bacteria

The antibacterial activity was measured using the agar well diffusion technique^28^. Using the Agar well diffusion technique, one milliliter of bacterial suspensions was spread onto each MHA plate. Punching wells (5 mm diameter) in the plates was done with a sterile stainless steel borer. Probiotics bacterial strains were cultivated in nutritional agar broth for 24 h at 28 °C overnight. After a 15-min incubation period, cells were extracted by centrifugation at 10,000 rpm for 15 min. The supernatant was collected and filtered through 0.22 mm thick membranes. A total of 50 l of filter sterilised supernatant was added to each well. After 24 h of incubation at 28 °C, the diameter of the inhibitory zone surrounding the well was determined in millimetres. All of the experiments were carried out twice.

### Antagonistic activity

Lechevalier and Waksman's Cross Streak Method^29^ were used to determine antagonistic activity. On the nutrient agar plate, a putative probiotic bacterium was streaked and cultured for 4 h at room temperature. The test organisms were then streaked to probiotic bacteria in a nutrient agar plate at 90 degrees. After the inoculation plates had been incubated at 28 °C for 48 h, the zone of inhibition was measured.

### Pathogenicity test

Immersion assays were used to assess the pathogenicity of the most efficient probiotic strains. The experiment was conducted out in triplicates in 500-l circular tanks with a temperature of 28°C and a salinity of 28ppt of water. *Mugil cephalus* (30 fish weighing 20–30 grammes) was used in the challenge test for healthy fish against probiotic microbes. The probiotic strain was cultivated for 24 h in tryptone soy broth. Cells were extracted by centrifugation at 6000 rpm for 20 min at 4 °C in a cooling centrifuge (Remi, Mumbai, India). After being washed, the cells were resuspended in sterile saline. After 48-h incubation at 28°C, the number of cells was estimated using spread plating on tryptone soy agar (TSA; Hi-Media). The residual probiotic cells in the sterile saline were added to the fish tanks at a concentration of 10^7^ cells/ml. The fish in the control aquariums were given a germ-free sterile saline solution. Fish were fed 5% of their body weight twice a day by hand (35% protein). At the conclusion of the trial, gross pathological features, abnormalities, and death rates were reported.

### Preliminary identification of the isolates

Gram staining and catalase tests were carried out on using bacteria for various morphological characteristics. Only Gram-positive and catalase-negative colonies were subcultured in Nutrient agar. For each colony, glycerol stocks (50% v/v) were made and kept at a temperature of 80 °C.

### Molecular characterization of isolated organisms by 16S rDNA sequencing

The 16S rRNA gene was amplified using PCR (thermal cycler). To amplify 1.5 kb DNA segments, the primers 27F (5-AGAGTTTGATCCTGGCTCAG-3) and 1492R (5-GGTACCTT GTTACGACTT-3) were utilized^30^.

### Sequencing and phylogenetic analysis

The amplified PCR products were purified using a Genei PCR purification kit (Genei, Bangalore). The amplified 16S rRNA gene was sequenced almost completely using an automated sequencer (Bioserve, Hyderabad). The sequence was altered with Clustal X mega software, and the amplified sequence's nearest neighbour was discovered using a BLAST search in the National Center for Biotechnology Information (NCBI) database. The sequencing findings were used to conduct homology searches. The phylogenetic tree was inferred using the neighbor-joining technique. The nucleotide sequences of the found species' partly complete 16S rRNA sequences were included in the Genbank database.

### Sample collection

Healthy fish (*Mugil cephalus*) weighing 20–30 grammes were collected from the Vellar estuarine of Portonovo, Cuddalore district, Tamil Nadu. Fish were transported to the wet lab and kept for 15-day acclimatisation period. In a circular plastic tank (200 l) filled with UV-treated estuary water and aeration, fish were separated into stocks at random. During this time, the fish were fed commercial pellets (4% body weight) twice a day. To maintain the water quality good, 20% of the water in the tanks was replaced every day with UV-treated estuarine water. The water temperature was maintained at 28 °C, salinity 28 ± 2 ppt and pH 8 °C ± 0.3 and dissolved oxygen content at 5.80.5 (mg/l) throughout the experiment. The levels of ammonia and nitrite in the water were maintained to a minimum.

### Probiotic diet preparation

*Bacillus subtilis* PRBD09 (KF765648) was cultured in tryptic soy broth at 28 °C and 150 rpm in a shaking incubator. The cell culture was suspended in sterile, normal saline solution before use after washing and centrifuging three times at 5000 rpm, 4 °C for 15 min. The integrity and uniqueness of the culture were constantly probed during the investigation. Fresh *B. subtilis* PRBD09 cells were well mixed with commercial feed (1:3), spread out, and left to dry at room temperature. The feed was then stored in clean plastic bags at 4 °C until it was needed. Two times a week, fish food was prepared. Each batch was tested for *B. subtilis* PRBD09 and total bacterial count*.* The control group did not receive probiotics, but *B. subtilis* PRBD09 was administered in feed at a concentration of 10^7^ cells/g for 15 days before the research animal was exposed to *A. hydrophila* in *Mugil cephalus*^31^.

### Bacterial pathogens

The Microbial Type Culture Collection (MTCC) in Chandigarh, India, provided A. hydrophila (MTCC 646). The bacteria were inoculated onto tryptone soy broth (HiMedia, Mumbai) and incubated at a temperature of 28 °C. The culture was centrifuged at 5000 rpm for 15 min at 4 °C. The cell pellets were washed, and the required dosage was prepared in PBS.

### Challenge test

15 days following the feeding, fish (20–30 g) were divided into four experimental groups at random. T1 received a basal diet, T2 received a basal diet plus an intra-peritoneal injection of 0.1 ml of fresh culture suspension containing 1108 CFU of *A. hydrophila* (T2), and T3 received a basal diet plus an intra-peritoneal injection of 0.1 ml of fresh culture suspension containing 1 × 10^8^ CFU of *A. hydrophila* (T3)^32^. Probiotic cells supplemented with basic meals were given to the fourth group of fish (T4), as well as an intra-peritoneal injection of 0.1 ml of fresh culture solution containing 2107 bacteria/ml of *A. hydrophila*. Survival, growth performance, and immunity indices were assessed every week for 30 days following infection.

### Growth parameter

The fish were starved for 24 h during the experiment. After being extensively anaesthetized (3-aminobenzoic acid ethyl ester, MS 222; 100 mg/ml), the fish were numbered and weighed. Based on the weight of each fish and the number of fish, the following formulae were used to calculate the specific growth rate (SGR), feed conversion ratio (FCR), and survival. Gain in body weight: Initial fish weight − Final fish weight (g)^33^, SGR = 100 [natural log of end weight – natural log of beginning weight]/experiment length^34^, FCR = weight growth/feed supplied (dry weight) (g)^35^.

### Non-specific immune response in *Mugil cephalus*

Determination of hematological parameters of Red blood cell and White blood cell count were determined using a haemocytometer with Neubauer counting chamber^36^. The hematocrit value was examined using the microhematocrit method and expressed as a percentage (% Ht)^37^. Nitrobluetetrazolium (NBT) assay followed the methods of Anderson and Siwick, NBT tests were used to assess the generation of oxidative radicals by neutrophils in blood during the respiratory burst^38^. The activity of lysozymes activity was assessed using a modification of a previously published turbidimetric method. 50 µl serum was placed in triplicate in a 96 well plate with 50 µl PBS and maintain the pH 5.8. After mixing, the serum was serially diluted till the last well. Finally, 50 l of sample was removed from the last well. Each well received 125  µl of *M. lysodeikticus*. The absorbance at 450 nm was measured at room temperature in an ELISA reader from 0 to 15 min. The lysozyme activity was converted to lysozyme concentration using hen egg white lysozyme as a reference^39,40^.

### Disease resistance and survival experiment

The disease resistance and survival experiment was carried out using the methodology of Harikrishnan et al.^41^ with slight modifications. A pathogenic strain of A. hydrophila was introduced into nutritious broth and incubated at 28 °C for 24 h. The culture was centrifuged at 5000 rpm for 15 min at 4 °C. The packed cells were washed, and the required dose was prepared using PBS. On the first day, probiotic-enhanced feed was fed in triplicate to groups of 15 fish at 4% of body weight. After 10 days of medication, the fish were challenged with A. hydrophila (6105 cfu/ml). After being exposed to the illness, the fish were checked for clinical symptoms and death.

### Culture conditions and strains

*Bacillus subtilis* PRBD09, a probiotic strain, was grown in 2 l Erlenmeyer flasks at 28°C, agitated at 200 rpm, and collected after 12 and 24 h in 500 ml of Luria–Bertani (LB) broth.

### Purification of extracellular protein

Scopes^42^ reported that remove the extracellular protein from the culture supernatant using ammonium sulphate precipitation followed by dialysis and gel filtration chromatography. The ammonium sulphate crystals were saturated in the supernatant until it reached 70% saturation. The aggregated proteins were then dialyzed for 20 min at 4 °C against buffer dialysis (10 mMTris-HCl pH 8.5], 100 mM NaCl, 10% Glycerol) using DO405-10 ft seamless cellulose tubing with 10,000 MWCO at 8000 rpm. The dyalisate was loaded onto a sephadex G-50 gel filtration column that had been pre-equilibrated with 0.2 M NaCl and 20 mM Tris–HCl (pH 7.5) buffer. All purification operations were completed at room temperature, with the exception of centrifugation. Elution was carried out using natural gravity, with fractions being collected every 2 min.

### Antibacterial activity of the fractions

Schillinger and Luke's agar well diffusion method^43^ was used to examine the fractions' antibacterial activity. Ampicillin was used as a positive control. The next day, the plates were checked for the presence of inhibitory zones, and the zones were measured. The experiment was carried out three times, and the mean results and standard error were calculated each time. The active antibacterial fraction was purified further using sephadex G-50 gel permeation chromatography. The antibacterial activity of the purified protein sub fraction was investigated once again, and the protein concentration in the active sub-fraction was evaluated using the Bradford technique^44^.

### Separation and purification of protein using SDS-PAGE

Following purification, the fractions were separated using the modified Laemmli technique^45^ to determine protein purity by visualising protein and activity bands on sodium dodecyl sulphate polyacrylamide gel electrophoresis (SDS-PAGE) and visualising protein and activity bands on SDS-PAGE. The gel band was stained using colloidal Coomassie staining. The gel was dyed with colloidal Coomassie after electrophoresis. After 4 h in the staining solution, the staining was removed with sterile distilled water. The protein bands were recorded, photographed, and maintained.

### Molecular characterization

Matrix-assisted laser desorption/ionization time of flight mass spectrometry was used to characterise extracellular proteins (MALDI-TOF MS). Protein spots were extracted from the gel and put in a gel digested with trypsin based on mass spectrometric sequencing of proteins from Coomassie-stained polyacrylamide gels.

### Washing of gel pieces

The gel fragments were treated for 15 min with a 50 mM ammonium bicarbonate/acetonitrile 1:1 v/v solution after being washed with water. All remaining liquid was drained away, and enough acetonitrile was poured to cover the gel fragments completely. It was decided to allow the gel plugs to shrink and stay together. The gel fragments were rehydrated in 50 mM ammonium bicarbonate after being removed from the acetonitrile. After 5 min, a 15-min incubation period was added with an equivalent amount of acetonitrile. After that, a little quantity of acetonitrile was applied to the gel fragments and allowed to shrink. The gel pieces were dried in a vacuum centrifuge after the acetonitrile was removed.

### Reduction and alkylation

The gel fragments were then allowed to swell in a freshly prepared solution of 10 mM dithiotreitol/50 mM ammonium bicarbonate. It was then incubated at 56 °C for 45 min. The tubes were then brought to room temperature. The surplus liquid was quickly drained and replaced with the same amount of freshly prepared 55 mM iodoacetamide in 50 mM ammonium bicarbonate, which was incubated in the dark for 30 min at room temperature. The solution of iodoacetamide was removed from the equation. The gel fragments were washed with 50 mM ammonium bicarbonate and 1:1 v/v acetonitrile for 15 min. After that, enough acetonitrile was poured over the gel fragments to completely cover them, and they were left to shrink. The gel pieces were dried in a vacuum centrifuge after the acetonitrile was removed.

### In-gel digestion

Using a newly produced enzyme solution, cover the gel pieces (25–50 ng enzymes, trypsin in 25 mM ammonium bicarbonate). It was then incubated at 37°C for 30 min. The excess enzyme solution was removed, and 25 mM ammonium bicarbonate was added to maintain the gel moist overnight.

### Extraction of peptides

Extraction buffer (50% acetonitrile/0.1% trifluoroacetic acid) was applied to the gel plugs and incubated for 30 min. After that, a 30-min extraction with 70% acetonitrile/0.1% trifluoro acetic acid was performed, followed by a final extraction with 100% acetonitrile/0.1% trifluoro acetic acid. The extracts were combined, vacuum dried, and then reconstituted with 10 mL MS grade water.

### MS analysis and protein search

The sample was combined with a ration of one:one (1:1) HCCA matrix and sprayed onto a polished steel target plate before drying. The Ultraflexreme MALDI TOF/TOF (BrukerDaltonics) was used to analyse it in reflectron positive mode 700–3500 Da. The device was calibrated with a standard peptide mixture before the sample analysis (BrukerDaltonics). In Ultraflexreme MALDI TOF/TOF, the parent mass was selected and fragmentation was performed using the lift method (BrukerDaltonics). The Mascot server in Swissprot was used to do the protein search.

### Mass fingerprinting of peptide

Peptide mass fingerprinting (PMF) by MALDI-MS and tandem mass spectrometry sequencing are used to identify proteins. This method generates a "mass fingerprint" of a protein that has been enzymatically digested using a sequence-specific protease like trypsin. This set of masses, which is frequently collected via MALDI-TOF/TOF, is compared to the theoretically expected tryptic peptide masses for each entry in the database. Proteins can be ranked based on the number of peptide matches. In order to calculate a match's level of confidence, more complex scoring algorithms incorporate mass accuracy and the fraction of the protein sequence covered^46,47^. PMF has been the most extensively used approach in proteomics. The Mascot server was used to analyse mass spectrum data. Mascot finds the most likely matches by comparing the observed spectra to a database of known proteins.

### Probability based scoring

Mascot uses a probabilistic scoring method to choose the best. This makes it possible to apply basic criteria to judge whether or not a finding is significant. Matches made on the basis of mass values are always considered probabilistic. The total score indicates how likely it is that the observed match is a coincidence. They cover a wide range of magnitudes and the fact that a "high" score correlates to a "low" likelihood, which might be perplexing. As a consequence, multiply the score by − 10*LOG10 (P), where P stands for absolute probability. As a result, a score of 10–20 turns into a score of 200.

### Protparam

Protparam (http://www.expasy.org/tools/protparam.html) estimates the many physicochemical properties that a protein sequence can provide. Protparam calculates molecular weight, extinction coefficient, instability index, theoretical pI, and amino acid composition, among other metrics. The theoretical pI and molecular weight were derived computationally, while the amino acid and atomic compositions are self-explanatory. Using the primary structural data, percentages of residues were calculated.

### Statistical analysis

Each experimental parameter's values were presented as arithmetic mean (AM) standard deviation (SD) using one-way ANOVA, with a probability of p 0.05 regarded significant. The influence of the probiotic diet on haematological and immunological markers was statistically analysed using Origin 6.1.

## Results

### Isolation of culturable intestinal microbiota

Totally Twenty seven (G1-G27) bacteria isolated was obtain in the intestine of fish *Mugil cephalus*. The isolated organsims separated by different morphological characters. 27 isolated were investigated for further investigated.

### Disk diffusion technique for antibacterial activity

**Figure 1:**
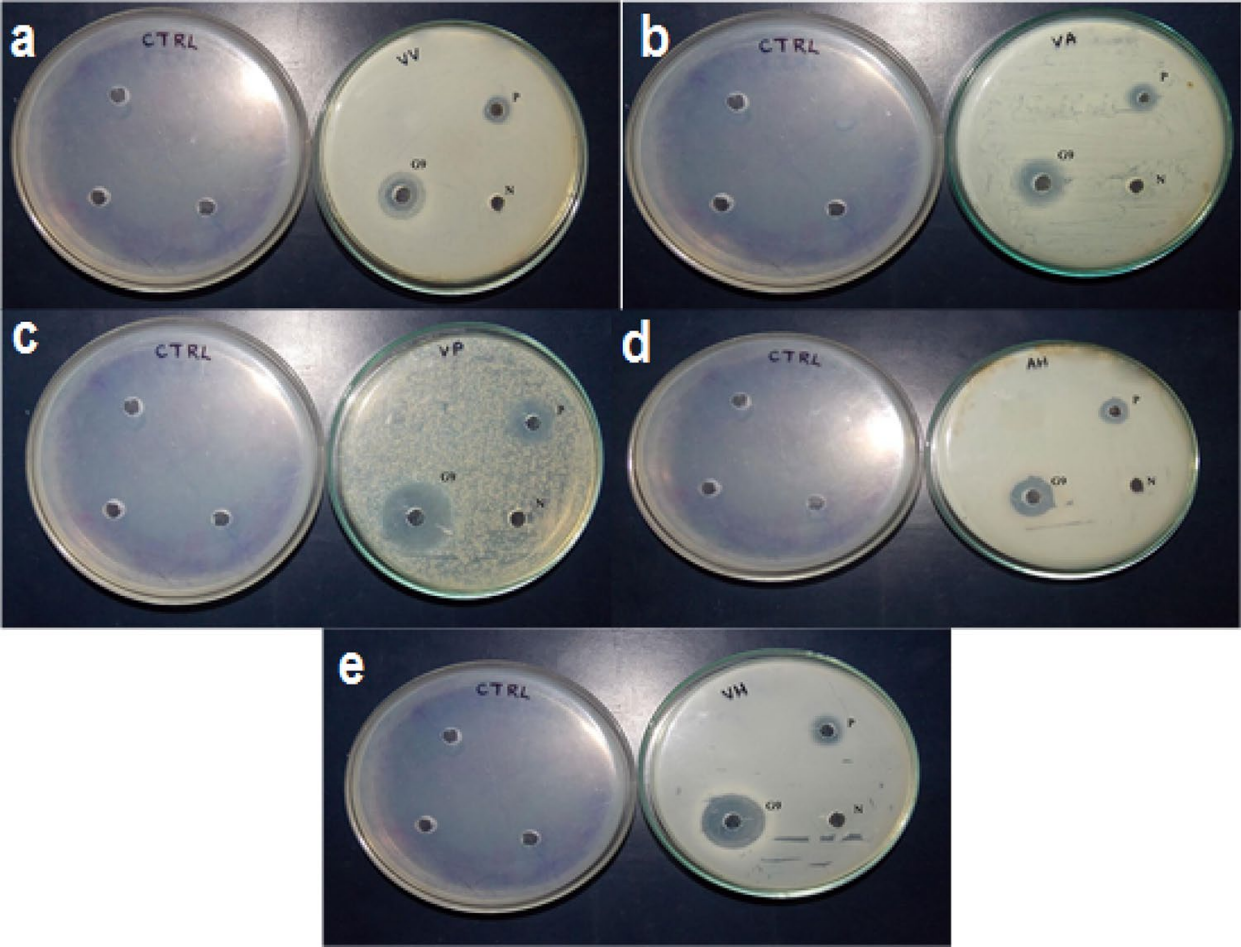
Antimicrobial activity of the G9 strain against test pathogen. VV: *Vibrio vulnificus* (MTCC1145), VA: *Vibrio alginolyticus* (MTCC 4182), VP: *Vibrio parahaemolyticus* (MTCC 451), AH: *Aeromonashydrophila* (MTCC 646) and VH: *Vibrio harveyi* (MTCC 3438).

The antibacterial activity of the 27 bacterial strains isolated from the gut of *Mugil cephalus* showed different zone of restraint against various fish pathogens such as *such as A. hydrophila, V. alginolyticus, V. harveyi, V. parahaemolyticus, V. vulnificus* (Table 1). G9 and G1 had the highest zone of restraint (20.60.5 and 151.15 mm) against *V. vulnificus*, whereas G3, G13, G14, and G18 had the lowest (4.60.5, 6.31.15, 6.31.15, and 6.60.57 mm). The highest zone of restraint in *V. alginolyticus* was found in G9 and G20 (20.31.5 and 13.61.52 mm), whereas the lowest zone of inhibition was found in G23, G13, and G14 (5.31.15, 6.31.15, and 6.30.5 mm). *V. parahaemolyticus* was more sensitive to G9, G15, and G20 (18.61.52, 12.31.15, and 13.61.15) and less sensitive to G3, G7, G12, G13, and G23 (7.61.15, 7.61.15, 7.61.15, and 7.31.15 mm). The largest zone of restraint in *A.hydrophila* was detected in G9 and G5 (181 and 14.62.5 mm), whereas the smallest zone of restraint was reported in G14, G11, and G3 (41, 4.330.57, and 51 mm). G9 and G20 (20.31.5 and 13.61.52 mm) had the biggest zone of restraint in *V. alginolyticus*, while G23, G13, and G14 had the smallest zones of inhibition (5.31.15, 6.31.15, and 6.30.5 mm). G9, G15, and G20 (18.61.52, 12.31.15, and 13.61.15) were more susceptible to *V. parahaemolyticus* than G3, G7, G12, G13, and G23 (7.61.15, 7.61.15, 7.61.15, and 7.31.15 mm). In *A. hydrophila*, the highest zone of restraint was found in G9 and G5 (181 and 14.62.5 mm, respectively), whereas the lowest zone of restraint was found in G14, G11, and G3 (41, 4.330.57, and 51 mm) (Fig. 1).

**Table 1:** Antibacterial activity of the isolates against fish pathogens.

S. no	Isolated organism	T1	T2	T3	T4	T5
1.	G1	15 ± 1.15	12 ± 1.15	12 ± 1	13 ± 2	12 ± 1
2.	G2	–	–	–	–	–
3.	G3	4.6 ± 0.5	7 ± 1	7.6 ± 0.5	5 ± 1	6.6 ± 0.5
4.	G4	12.3 ± 0.5	12.3 ± 1.5	10.6 ± 0.57	10.3 ± 0.5	11.6 ± 0.5
5.	G5	11.6 ± 0.5	12 ± 1	11.3 ± 0.5	14.6 ± 2.5	14.3 ± 1.15
6.	G6	11.3 ± 1.52	11 ± 1	12 ± 1	11.3 ± 1.5	11 ± 1
7.	G7	8.3 ± 1.15	10 ± 1	7.6 ± 1.15	8.33 ± 1.15	11 ± 1
8.	G8	11 ± 1	13.3 ± 1.52	12.6 ± 0.5	11.3 ± 1.5	11.3 ± 0.15
9.	G9	20.6 ± 0.5	20.3 ± 1.5	18.6 ± 1.52	18 ± 1	17.6 ± 1.15
10.	G10	9 ± 1.73	8 ± 1	8 ± 1	6.66 ± 1.52	8 ± 1
11.	G11	9 ± 1	8.3 ± 1.52	8.66 ± 1.52	4.33 ± 0.57	7.6 ± 1.15
12.	G12	12.3 ± 1.15	12.3 ± 1.15	12.3 ± 1.15	12 ± 1	11.6 ± 1.15
13.	G13	6.3 ± 1.15	6.33 ± 1.15	7.6 ± 1.15	6.33 ± 1.15	5.6 ± 1.15
14.	G14	6.3 ± 0.5	6.33 ± 0.5	7.6 ± 0.5	4 ± 1	6 ± 1
15.	G15	12.3 ± 1.5	12.3 ± 1.5	13 ± 1	11.3 ± 0.57	11.3 ± 0.57
16.	G16	11.3 ± 0.5	11.3 ± 0.5	11.3 ± 0.57	13 ± 1	12.6 ± 0.57
17.	G17	12.3 ± 0.57	12.3 ± 0.57	12.3 ± 0.57	12 ± 1	13 ± 1
18.	G18	6.6 ± 0.57	9.33 ± 0.57	9.6 ± 0.57	8 ± 1	8 ± 1
19.	G19	10 ± 1	11 ± 1	11 ± 1	11.6 ± 0.57	11.6 ± 0.57
20.	G20	12.3 ± 0.57	13.6 ± 1.52	13.6 ± 1.15	13	12.6 ± 0.57
21.	G21	–	–	–	–	–
22.	G22	12.6 ± 0.57	12.3 ± 0.57	14.3 ± 0.57	13.6 ± 0.57	12 ± 1
23.	G23	9.3 ± 0.57	5.3 ± 1.15	7.3 ± 1.15	6.6 ± 0.57	5 ± 1
24.	G24	11 ± 1	10.3 ± 0.57	10.6 ± 0.57	11 ± 1	13.3 ± 1.5
25.	G25	12.3 ± 1.5	12.3 ± 0.57	12.6 ± 0.5	13 ± 1	13 ± 1
26.	G26	12	11.3 ± 1.15	11.6 ± 1.52	11 ± 1	13.6 ± 1.15
27.	G27	11 ± 1	11.6 ± 0.57	11.3 ± 1.15	11 ± 1.15	12.6 ± 0.57

T1—*Vibrio vulnificus*; T2—*Vibrio alginolyticus*; T3—*Vibrio parahaemolyticus*; T4—*Aeromonas hydrophila*and; T5*—Vibrio harveyi.*

### Antagonistic activity

**Figure 2:**
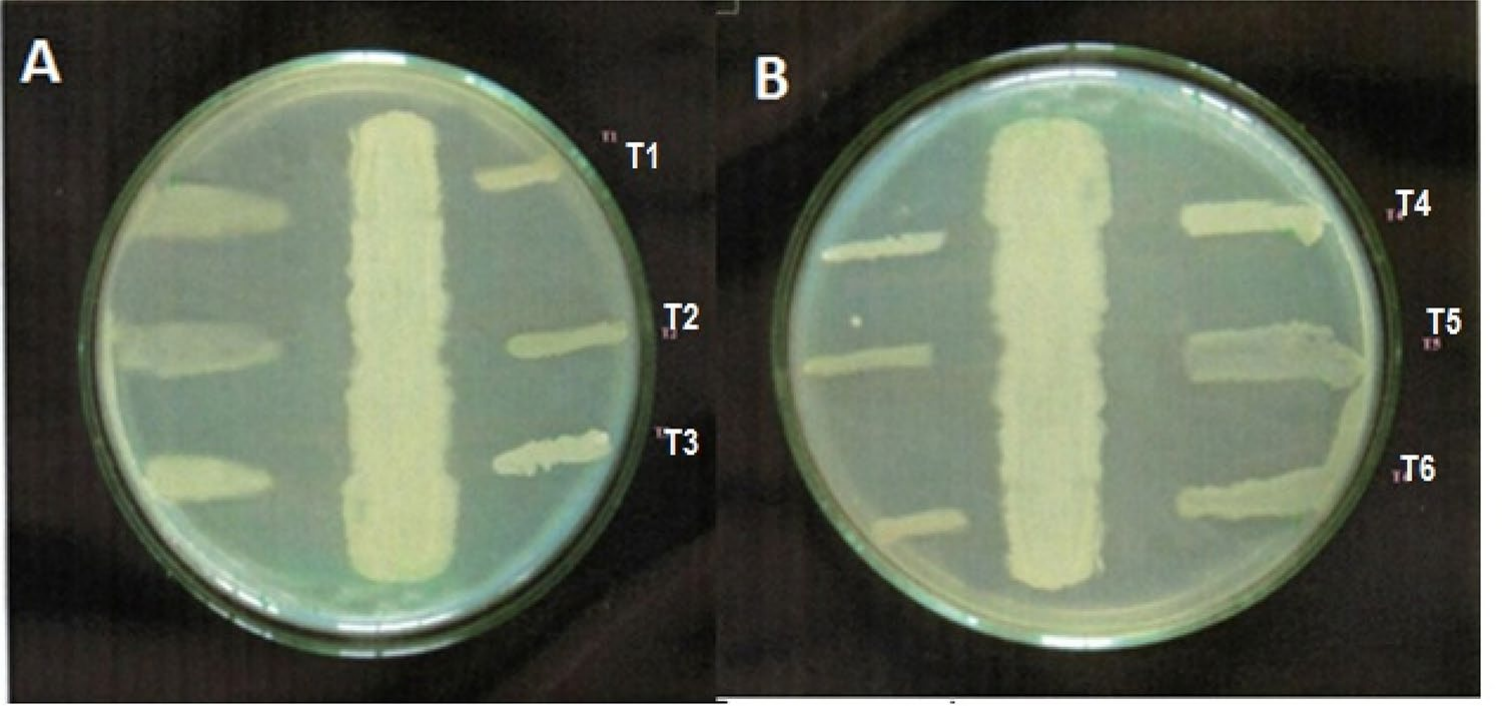
Antagonistic activity of strain G9 against test pathogen (**A**: T1—*Vibrio vulnificus*; T2—*Vibrio alginolyticus*; T3—*Vibrio parahaemolyticus*; **B**: T4—*Aeromonas hydrophila* and T5—V *Vibrio harveyi*).

The five strains (G8, G17, G25, G26, and G27) did not restraint the pathogenic test microorganisms using the cross streak technique. The strain G9 had an restraint zone higher than 10 mm against all of the test pathogens, followed by the strains G16, G5, G22, G15, and G24, all of which had an inhibition zone greater than 5 mm. Against the test pathogens, the inhibition zones formed by strains G1, G20, G12, G4, G6, and G19 were less than 5 mm (Table 2). G9 was chosen for further research because of its larger zone of restraint in the cross streak technique and strong antibacterial activity (Fig. 2).

**Table 2:** Inhibition patterns produced by the isolates in cross streak method.

S. no	Isolatedorganism	T1	T2	T3	T4	T5
1.	G1	+	+	+	+	+
2.	G4	+	+	+	+	+
3.	G5	++	++	++	++	++
4.	G6	+	+	+	+	+
5.	G8	−	−	−	−	−
6.	G9	+++	+++	+++	+++	+++
7.	G12	+	+	+	+	+
8.	G15	++	++	++	++	++
9.	G16	++	++	++	++	++
10.	G17	−	−	−	−	−
11.	G19	+	+	+	+	+
12.	G20	+	+	+	+	+
13.	G22	++	++	++	++	++
14.	G24	++	++	++	++	++
15.	G25	−	−	−	−	−
16.	G26	−	−	−	−	−
17.	G27	−	−	−	−	−

+++  > 10 mm ;++ 5–10 mm; +  < 5 mm; − no zone.

### Determination of pathogenicity for G9

Organisms G9 did not cause any abnormalities or death in *Mugil cephalus*, and the fish appeared to be healthy, proving their non-pathogenicity.

### Preliminary identification of the isolates

**Figure 3:**
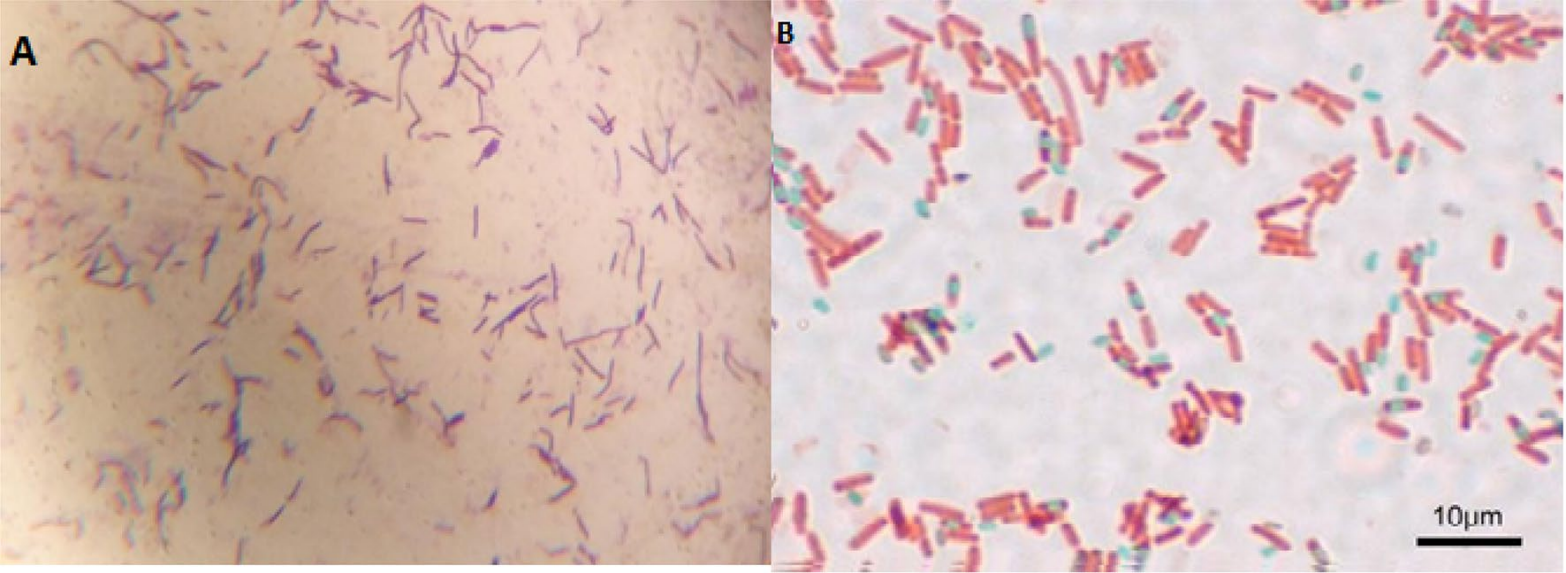
(**A**) Strain G9: Gram’s staining (Gram positive rod shaped bacteria) under oil immersion microscope. (**B**) Strain G9-Endospore staining (Rod shaped bacteria) under oil immersion microscope (Lycia 100X).

**Figure 4:**
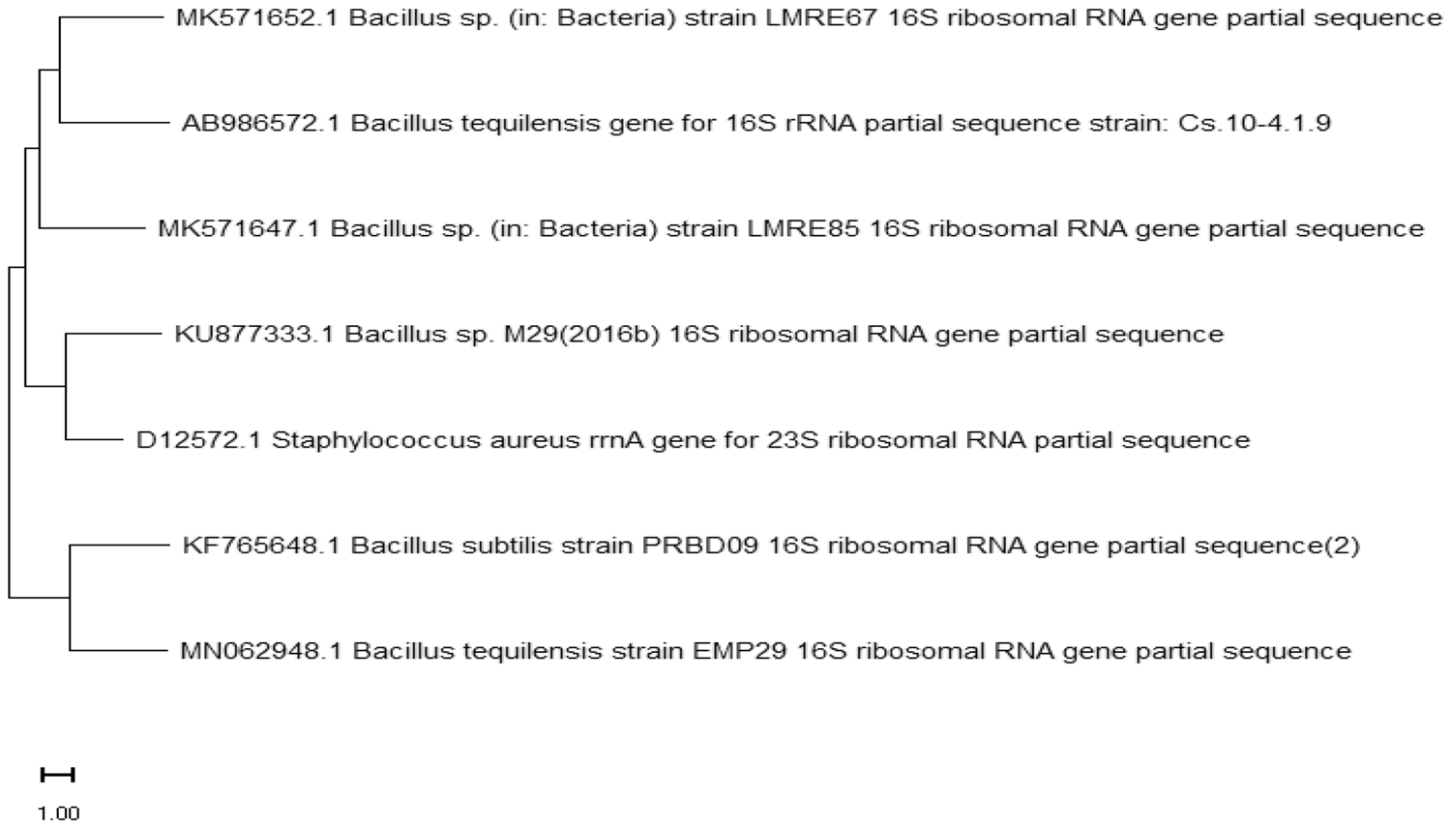
Neighbour-Joining (NJ) tree of 16S rRNA partial gene sequencing of probiotic Strain G9 -*Bacillus subtilis*PRBD09 and other taxa.

Morphology identification and Molecular characterization of the bacteria was carried out. For further Molecular characterization we done 16S rRNA gene sequence and Morphology identification we follow Gram-positive and spore staining colonies (Fig. 3A,B) were chosen. DNA was extracted from the bacteria and used for the PCR amplification of the DNA. After the PCR analysis PCR product were purified and it’s used for the16S rRNA sequencing. The sequencing was sent to the National Center for Biotechnology Information [NCBI], Bethesda, Maryland, USA, and the accession number for the G9 bacterium was assigned (KF765648). The bacteria's 16S rRNA gene sequence was determined using phylogenetic analysis using neighbour joining (NJ) methods. This research looked at eight different nucleotide sequences. The positions of the 1st + 2nd + 3rd + Noncoding codons were included. All uncertain places in each sequence pair were removed (pairwise deletion option). In all, 1422 locations were included in the final dataset. MEGA11 was used to undertake evolutionary analysis (Fig. 4).

### Growth parameter

For a 1-month period, Body weight gain, specific growth rates, condition factor, and survival rate were all significantly greater (p 0.05) in fish fed a basal diet supplemented with probiotic feed compare with fish fed an exclusively basal diet (control). The overall mean weight and specific growth rate (SGR) of the fish improved as the probiotic feed was increased after 35 days of feeding. The fish fed the baseline diet had the slowest development rate. Furthermore, the probiotic group's Feed conversion ratio (FCR) was lower than the control group's. In comparison to the control group, the survival rate was high (Table 3).

**Table 3:** Growth parameters of *Mugil cephalus.*

S. no	Studied group	Body weight gain (g)		Specific growth rate (g/day)	Feed conversion ratio (FCR)	Condition factor (K)
		Initial weight	Weight gain			
1.	Control	27 ± 0.86	5.8 ± 0.25	0.0046	5.8655	1.61 ± 0.03
2.	Probiotic	27.6 ± 1.42	12.3 ± 1.45	0.0096	4.7379	1.8 ± 0.007

### Non-specific immune parameters

**Figure 5:**
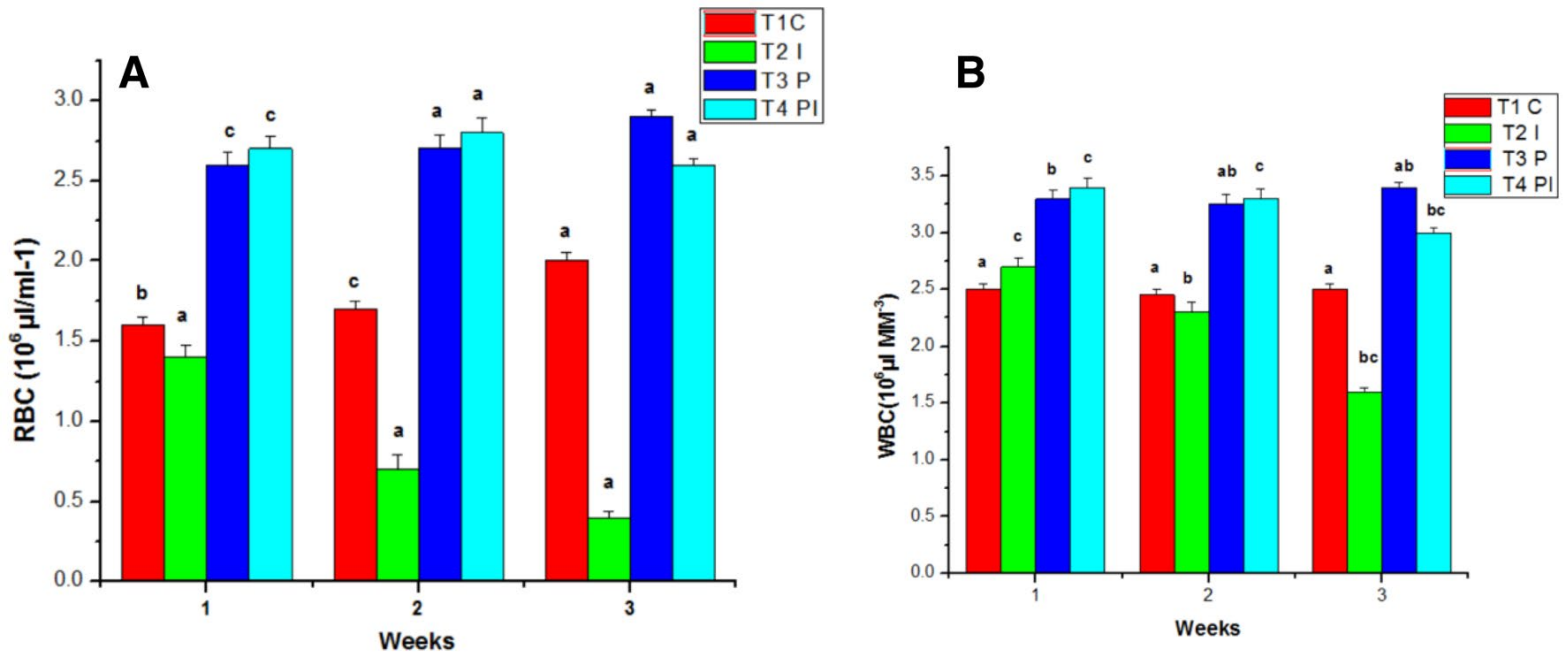
Effects of Probiotics on the number of (**A**) RBC and (**B**) WBC blood of *MugilCephalus*. Data are reported as means ± SD (n = 10). *Significantly different from control by unpaired Student t test (p < 0.05).

The Red blood cells count in the T1 group remained relatively constant during the trial, but the count in the T2 group declined from the first to the third week. In T3 and T4, the RBC count was considerably higher than in the control group. T4's RBC count was shown to be lower in the third week, whereas T3 had the highest RBC count among the treatment groups in the third week (Fig. 5A). Changes in the standard parameters engaged in the groups were identified during the experiment.

**Figure 6:**
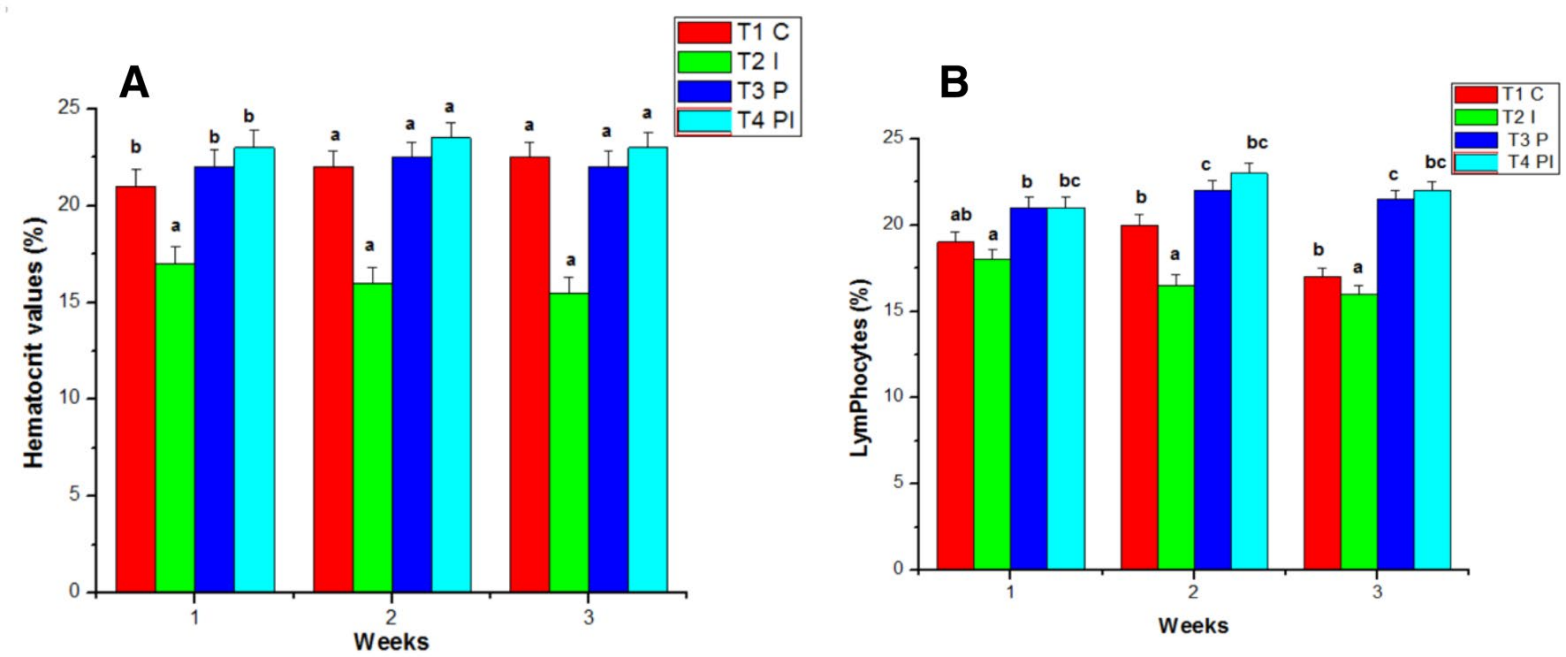
Effects of Probiotics on the number of (**A**) hematocrit values and (**B**) lymphocytrescount in blood of *Mugil cephalus*. Data are reported as means ± SD (n = 10). *Significantly different from control by unpaired Student t test (p < 0.05).

**Figure 7:**
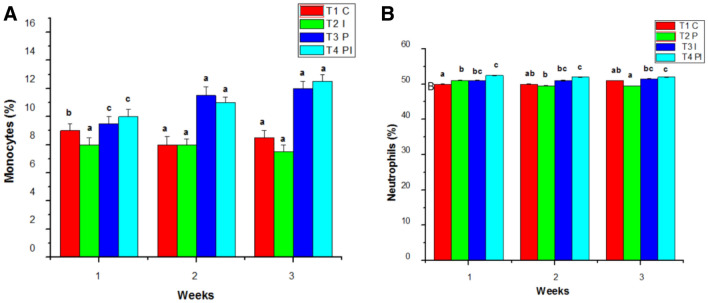
Effects of Probiotics on the number of (**A**) monocytes count (**B**) neutrophils count blood of *Mugil cephalus*. Data are reported as means ± SD (n = 10). *Significantly different from control by unpaired Student t test (p < 0.05).

**Figure 8:**
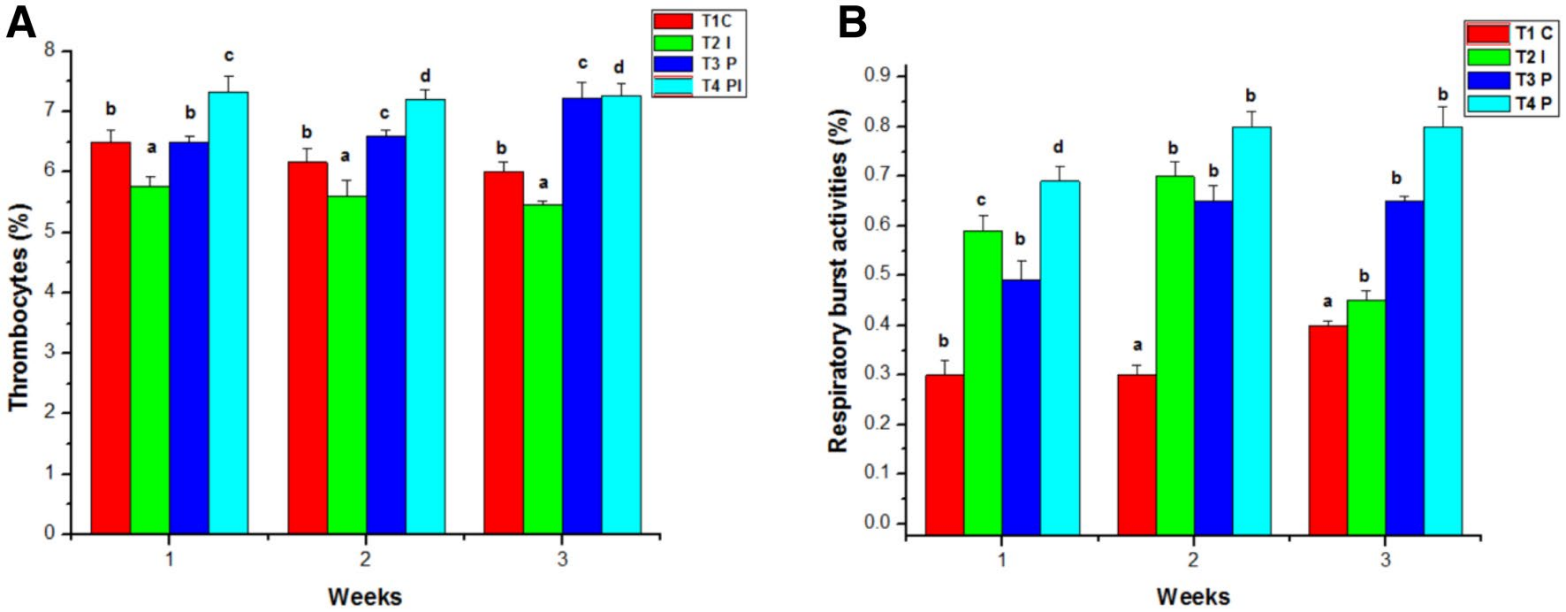
Effects of Probiotics on the number of (**A**) thrombocytes and (**B**) respiratory burst activities blood of *Mugil cephalus*. Data are reported as means ± SD (n = 10). *Significantly different from control by unpaired Student t test (p < 0.05).

**Figure 9:**
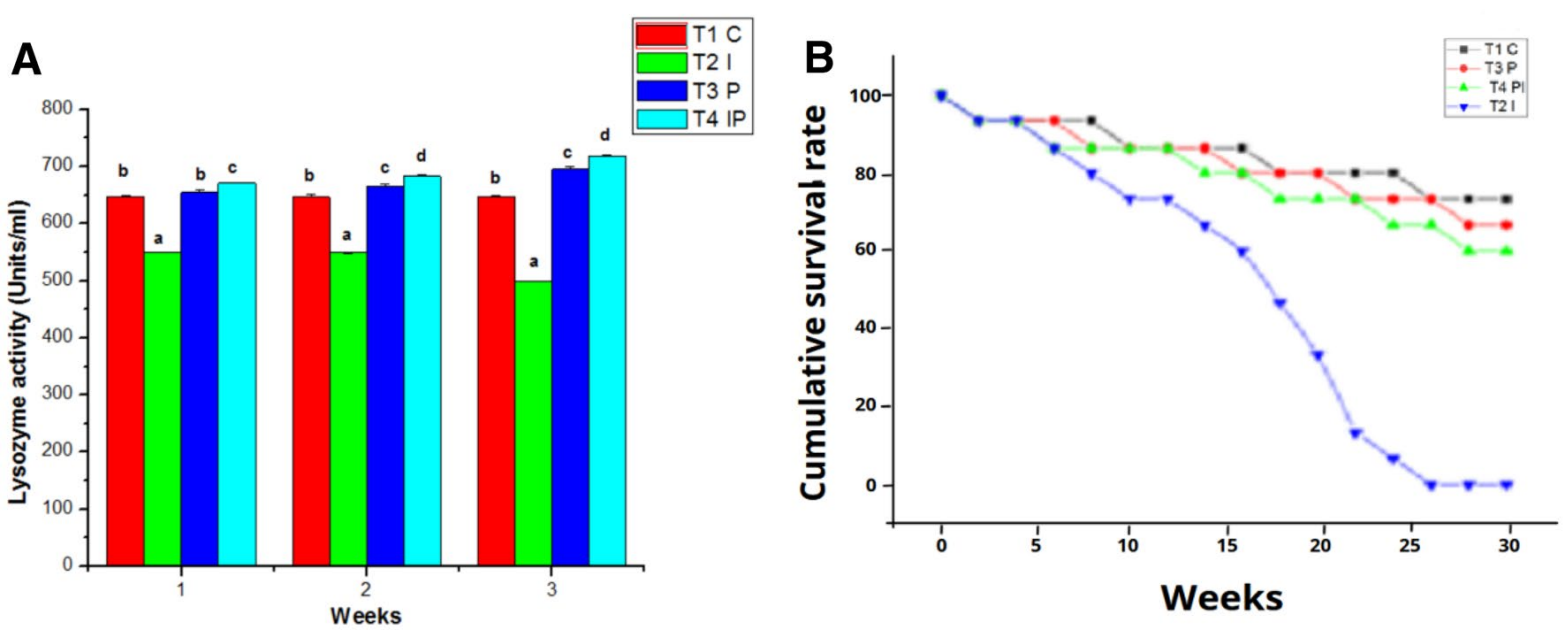
Effects of Probiotics on the number of (**A**) lysozyme activity blood of *Mugil cephalus*. (**B**) Cumulative survival rate of *Mugil cephalus during the experimental studies*. Data are reported as means ± SD (n = 10). *Significantly different from control by unpaired Student t test (p < 0.05).

The White blood count of the control group (T1) remains normal throughout the study. WBC counts in the infected group (T2) grew slightly in the initial week of the studies, then decreased in the second and third weeks. Similarly, the WBC count in T4 was considerably greater in the first week than in T3, followed by a decline in the counts in the second and third weeks of testing. T3 had the greatest blood count in the experimental group throughout the studies when compared to the control and other groups (Fig. 5B). In the control group (T1), the hematocrit (HT percent) was within normal ranges. Infected groups had a lower hematocrit percentage than the control group because of infection. The hematocrit readings in T3 and T4 increased considerably during the study (Fig. 6A). Total lymphocytes, monocytes (Fig. 6B), neutrophils, and thrombocytes were all significantly higher in T3. Infected fish given probiotic feed had a larger number of parasites than control fish. The total lymphocytes, monocytes (Fig. 7A), neutrophils (Fig. 7B) and thrombocytes (Fig. 8A) counts in T2 had fallen significantly from the first to the third week On compared to the control, the respiratory burst activity (Fig. 8B) of infected fish given a regular diet increased significantly. As a result of the probiotic diet, the diseased fish showed increased respiratory burst activity. The lysozyme activity of infected fish provided probiotic feed did not improve in the initial week, but it was greatly improved after the initial week in the infected fish (Fig. 9A).

### Disease resistant

The cumulative survival rate was used to determine disease resistance (Fig. 9B). The resistance of fish to pathogenic organisms *A. hydrophila* was tested in this experiment. The control fish died at a rate of more than 90% after being injected with *A. hydrophila*. The fish given the probiotic *B. subtilis*, on the other hand, fared better against pathogen infection, surviving between 88 and 100% of the time.

### Extracellular protein purification

More than a few studies have demonstrated that the high amount of protein secretion occurs between the end of the exponential phase and the beginning of the stationary phase. As a result, the *B. subtilis* cells were cultivated in an Luria broth (LB) medium and collected all over the late exponential phase in this series of studies. After then, the extracellular proteins were precipitated. At 70% ammonium sulphate saturation, maximum extracellular protein precipitation was observed. The pellets were dialyzed and placed on a G-50 (sephadex) column after precipitation. The powerful sub fraction was subsequently identified based on the high anti-bacterial activity. Using the Bradford test, the protein content in the bioactive purified sub fraction was determined to be 1.13 ± 0.05 mg/ml mg/ml.

### Sodium dodecyl sulphate–polyacrylamide gel electrophoresis (SDS-PAGE)

**Figure 10:**
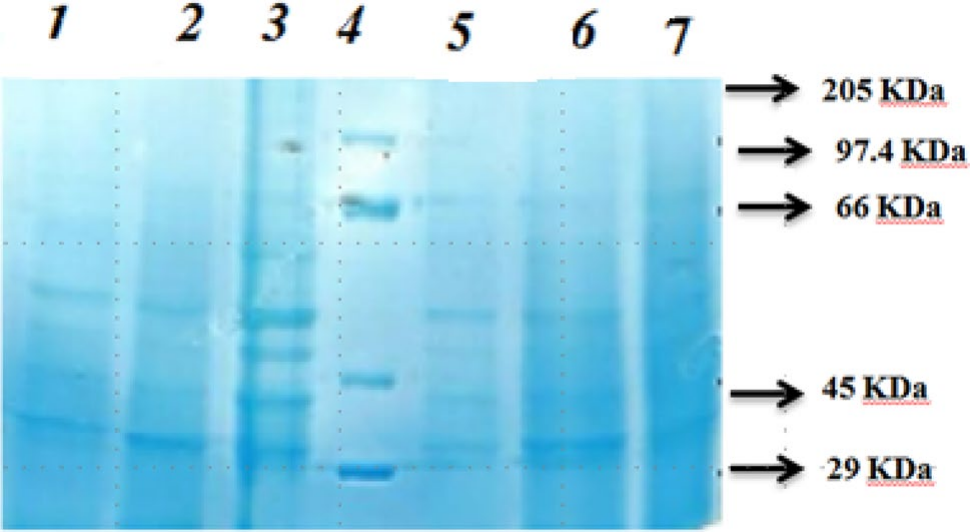
Quantification of protein by Bradford protein assay method sodium dodecyl sulphate–polyacrylamide gel electrophoresis.

SDS–PAGE was used to examine the bands in a purified sub fraction of extracellular protein from the potent probiotic bacteria *B. subtilis* PRBD09. The proteins' molecular weights were calculated, and the molecular weights of the five bands were found to range from 29 to 66 kDa (Fig. 10). Extracellular protein molecular characterization utilizing matrix aided laser desorption/ionization time of flight mass spectrometry (MALDI TOF MS MS). MALDI-TOF MS analysis was used to identify the amino acid sequences of the extracellular proteins. The mass-to-charge ratio is plotted on the X-axis, while the intensity is plotted on the Y-axis.

**Figure 11:**
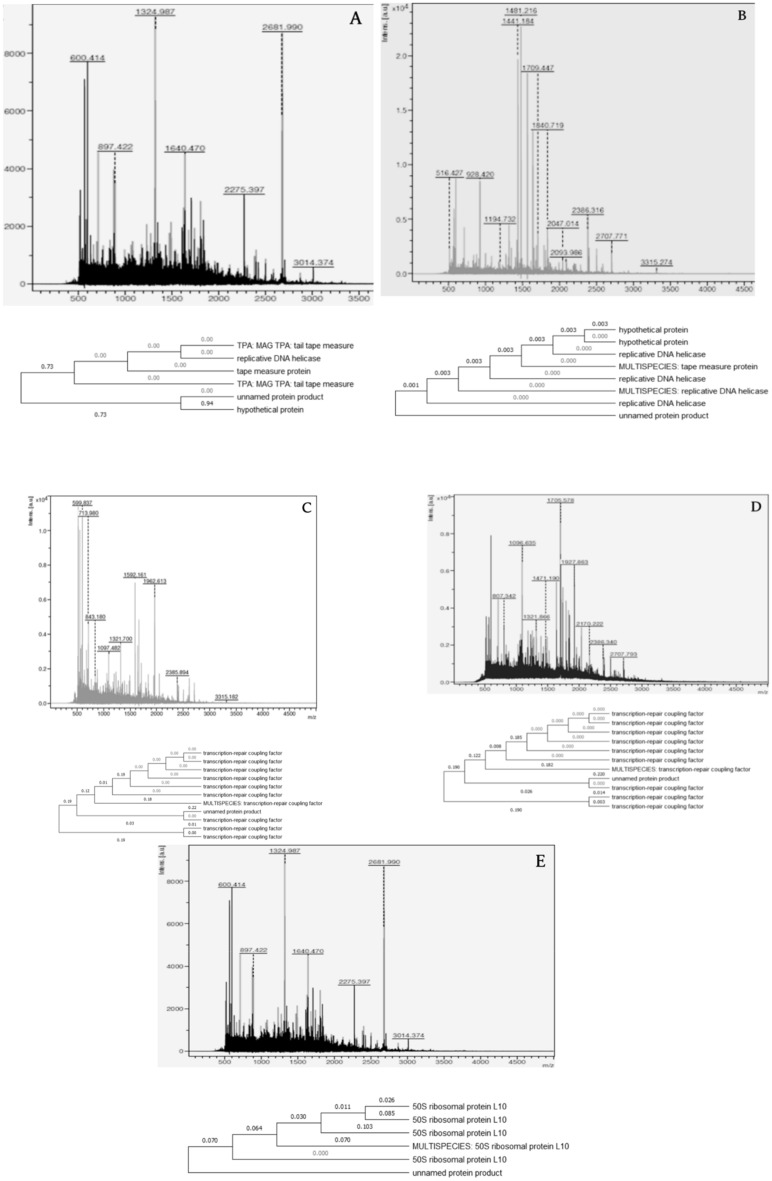
Peptide mass spectra of the tryptic digested peptides as obtained from MALDI TOF mass spectrometry (**A** Sample 8, **B** Sample 9, **C** Sample 10, **D** Sample 11 & **E** Sample 12).

In Fig. 11, the MALDI-TOF spectrum profiles of the five samples were marked sample 08, 09, 010, 011, and 012 (A–E). Table 4 shows the mass y charge ratio of the tryptic digested peptide fragments of the purified samples. For sample 08, the peaks 2275.397 and 3014.374 with mass by charge ratio of tryptic digested peptide fragment were selected, and the sequence was predicted to be KGIQNVTENTANLLASYINAIRA and KSMTELLLIKPSLSEN PIFEKHFSLLKN. Sample 08's full sequence was predicted to be KGIQNVTENTANLLASYINAIRA. KSMTELLLIKPSLSENPIFEKHFSLLKN. The peaks with mass by charge ratios of 600.370, 821.303, 1190.799, and 1691.365 were picked for sample 09, and the sequence was predicted to be RGWLPKG, KEEWTEKV, RKFLNGEILEKQ, and KRVSAYDEGGAYGYQRA. RGWLPKGKEEWTEKVRKFLNGEILEKQKRVSAYDEGGAYGYQRA was predicted for the complete sequence of sample09. The sequence RTHDVKSRLALAEAKKA and KVGSWRLENASLFVTLEPCPMCSGAMILSR were predicted from the peaks of sample 010 with mass by charge ratios of 599.837, 843.1802, and 33.15, respectively.

**Table 4:** Mass by charge ratio profile of the purified.

m/z	s/N	Quality factor	Resolution	Intensity	Area
**Sample 08**					
600.414	44.1	6025	3417	7439.29	2062
897.422	15.6	4914	4889	3940.24	1201
1324.987	41.6	41,440	5589	8833.50	5065
1640.470	26.1	24,873	5742	4504.00	3687
2275.397	34.1	44,855	7088	2420.47	2748
2681.990	174.4	50,713	7340	5866.57	8185
3014.374	17.3	10,162	6943	314.34	563
**Sample 09**					
600.370	55.4	5531	3954	9283.77	2239
821.303	18.6	5832	4539	3821.89	1239
1190.799	81.8	23,770	5578	14,517.46	6938
1321.808	13.2	11,229	5844	2138.09	1154
1691.365	127.1	90,472	6595	15,301.78	11,840
2386.235	21.7	12,564	7804	912.67	1005
2865.965	10.3	1867	5816	176.22	326
3315.211	6.4	1315	9355	52.08	84
**Sample 10**					
599.837	49.9	2123	3782	10,968.62	2764
713.980	18.9	2715	4024	4601.21	1382
843.180	6.5	1014	4609	1562.67	510
1097.482	13.6	6190	5627	2860.00	1168
1321.700	19.8	14,186	5890	3451.80	1878
1592.161	54.5	41,154	6735	7223.64	4841
1962.613	72.0	26,439	7266	6145.19	5431
2385.894	28.4	37,399	7316	1173.44	1368
3315.182	8.1	2052	8059	66.99	130
**Sample 11**					
807.342	12.2	1339	4377	2838.89	818
1096.635	29.8	4057	5095	6496.62	2756
1321.866	12.6	3085	5338	2771.62	1446
1471.190	7.7	1504	4866	1747.12	1104
1705.578	49.1	26,759	5677	8593.09	7329
1927.863	34.0	35,191	5827	5156.82	5242
2170.222	11.6	7278	5924	1478.82	1517
2386.340	13.5	6393	6052	1247.92	1553
2707.793	11.2	3946	5737	695.45	1032
**Sample 12**					
56.427	20.9	2516	2708	2344.38	669
928.420	55.5	23,096	4672	8654.07	3325
1194.732	7.6	2245	5067	1095.57	566
1441.184	154.1	75,230	5698	20,106.33	13,222
1481.216	184.0	100,723	5742	23,638.83	16,214
1709.447	32.4	58,858	6462	3710.67	3016
1840.719	39.1	70,791	6794	4077.76	3672
2047.014	16.0	18,525	7245	1218.42	1164
2093.986	15.6	21,980	5779	1107.06	1346
2386.316	90.3	19,463	6813	4074.35	5166
2707.771	73.7	19,401	7218	1901.18	2738
3315.274	30.1	1687	8873	297.13	537

RTHDVKSRLALAEAKKAK VGSWRLENASLFVTLEPCPMCSGAMILSR was predicted for the complete sequence of sample 010. The sequence was expected to be KLVLDSTIGRGYKY, KNSYGVGVERIFPLHSPRI, and KADMESDRPMDRLICGDVGFGKT based on the peaks of sample 011 with mass by charge ratios of 1321.866, 1927.863, and 2386.340. KLVLDSTIGRGYKYKNSYGVGVERIFPLHSPRIKADMESDRPMDRLICGDVGFGKT was predicted for the complete sequence of sample 011. The sequences KLKELGLQKN, KCWDEIISQAKMLSKA, RGLVTVLSGPSRGLVTCLSQLAEKK, and MLQSYEAIYENGQVKWLGEQPQVKS were predicted from the peaks of sample 012 with mass by charge ratio of tryptic digested peptide fragments of 928.420, 1709.447, 2386.316, and 2707.771 respectively. KLKELGLQKNKCWDEIISQAKMLSKARGLVTVLSGPSRGLVTCLSQLAEKKMLQSYEAIYEN GQVKWLGEQPQVKS was predicted for the complete sequence of sample 012 (Fig. 11A–E).

### In silico analysis

The acquired sequence was subjected to in silico analysis. The peptide sequence was predicted, and the five extracellular proteins' peptides were identified using a MASCOT search and a homology search in NCBI BLASTp. The putative protein, cytidinedeaminase transcription repair coupling factor, and Sir2 protein were expected to be found in samples 08, 09, 010, 011, 012.

### Protparam

The physico-chemical properties of a protein sequence are calculated using ProtParam. Table 5 shows the physicochemical properties of the predicted extracellular proteins. Table 6 shows the amino acid makeup of the five samples as a percentage. Sample 08's predicted peptide sequence was 51 amino acids long, with asparagine accounting for 11.8% of the total. Sample 09 contained a 44-amino-acid projected sequence, with glutamine and isoleucine accounting for 13.6% of the peptide sequence. Sample 010's predicted sequence was 47 amino acids long, with alanine accounting for 12.8% of the peptide sequence. Sample 011's anticipated sequence was 56 amino acids long, with glycine accounting for 12.5% of the peptide sequence. Sample 012's projected sequence was 76 amino acids long, with leucine accounting for 14.5% of the peptide sequence.

**Table 5:** Amino acid composition using protparam tool.

Amino acid	08%	09%	010%	011%	012%
Alanine (A)	7.8	6.8	12.8	1.8	5.3
Arginine (R)	2.0	9.1	8.5	8.9	2.6
Asparagine (N)	11.8	2.3	2.1	1.8	2.6
Aspartic acid (D)	0.0	0.0	2.1	8.9	1.3
Cysteine (C)	0.0	4.5	4.3	1.8	2.6
Glutamine (Q)	2.0	13.6	0.0	0.0	9.2
Glutamic acid (E)	7.8	0.0	6.4	3.6	7.9
Glycine (G)	2.0	2.3	4.3	12.5	7.9
Histidine (H)	2.0	6.8	2.1	1.8	0.0
Isoleucine (I)	9.8	13.6	2.1	7.1	3.9
Leucine (L)	15.7	0.0	12.8	7.1	14.5
Lysine (K)	9.8	2.3	8.5	8.9	13.2
Methionine (M)	2.0	2.3	4.3	3.6	2.6
Phenylalanine (F)	3.9	2.3	2.1	3.6	0.0
Proline (P)	3.9	2.3	4.3	5.4	2.6
Serine (S)	9.8	4.5	10.6	7.1	9.2
Threonine (T)	5.9	6.8	4.3	3.6	2.6
Tryptophan (W)	0.0	4.5	2.1	0.0	2.6
Tyrosine (Y)	2.0	0.0	0.0	5.4	2.6
Valine (V)	2.0	0.0	6.4	7.1	6.6

**Table 6:** Physico- chemical characteristics predicted by ExpasysProtParam Tool.

Physiochemical parameters	08	09	010	011	012
Length	51	44	47	56	76
Molecular weight	5714.6	5172.8	5173.1	6288.2	8506.9
Theoretical pI	9.31	9.46	9.69	9.30	9.43
Extinction coefficient at 280 nm	1490	15,470	5625	4470	14,105
Instability index	10.28	35.81	19.23	12.39	49.07
Aliphatic index	112.94	55.45	89.36	78.21	96.18
Grand average of hydropathicity (GRAVY)	0.096	1.252	0.049	0.457	0.378
Number of negative residues –R	4	7	4	7	7
Number of positive residues +R	6	10	8	10	12

## Discussion

The effects of using probiotics in fish farming were investigated by Nayak and Qi et al.^48,49^. Probiotics have been found to improve growth performance^50^, disease control through immunological strengthening, and pathogen exclusion in fish aquaculture^51,52^. Microorganisms thrive in the intestine of fish, with densities that are significantly higher than in the surrounding water^53^. One of the most sought-after features in putative probiotics is antibiotic action or infection suppression. Lysozymes, proteases, siderophores, hydrogen peroxide, and bacteriocins are chemicals produced by probiotic bacteria that exert bacteriostatic or bactericidal effects on pathogenic pathogens^54^.

The effects of using probiotics in fish farming were investigated by Nayak and Qi et al.^48,49^. The use of probiotics in fish farming has proven to be beneficial. Several potential probiotics have been found in aquaculture to produce antibacterial compounds that restrict pathogenic microbe development while maintaining gut microecological balance^55^. As a result, many aquaculture probiotics have been shown to have direct antibacterial effects against pathogens^56^. In this study, strong probiotic bacteria were isolated from *Mugil cephalus*' digestive tract, revealing a diverse diversity of bacterial strains. Microorganisms have also been found to provide 15–30% of the organic material in mullet stomachs. Because the gut content of mullets contains a substantial amount of plant matter, the existence of such a diverse community of bacteria suggests that they may play a role in the decomposition of plant materials^57^.

Moriarity^58^ conducted similar research on the intestinal composition of mullets. He discovered that *Mugil cephalus* had a high level of muramicacid in its stomach, indicating that it had consumed microorganisms connected with sediments, thereby forming a significant component of the detritus-based food chain. He also stated that both gramme positive and gramme negative bacteria exist in the digestive tract's microbial community. Disease outbreaks in fish culture systems pose a significant risk to fish farmers in terms of financial loss. Antibacterial chemicals used indiscriminately have resulted in the emergence of resistance strains of fish bacterial infections^59^. *Antibiotic resistance has been discovered in A. salmonicida, A. hydrophila, V. anguillarum, V. vulnificus, V. alginolyticus, V. parahaemolyticus, V. harveyi, P. fluorescens, P. piscida, and Edwardsiella tarda*^60^*.* According to clinical evidence, antibiotic-resistant strains of fish pathogens have developed over the time that antibiotics have been used to reduce fish sickness*. V. anguillarum, V. vulnificus, V. alginolyticus, V. parahaemolyticus, V. harveyi,* and *A. hydrophila* were investigated for antibacterial and antagonistic action against five common fish pathogen*.* Against the fish pathogens, 17 of the 27 isolates had a zone of inhibition higher than 10 mm. The isolates from halibut larvae had a similar frequency of inhibitory bacteria^61^ followed by Brunt and Austin^62^ in rainbow trout^63^ and in shrimp^49,64^. The production of chemical compounds having bactericidal or bacteriostatic actions was said to be the cause of the inhibition.

In the cross streak method, the strain G9 showed a wide range of inhibitory activity against the test pathogens. G9 created an inhibitory zone against the pathogenic *V. harveyi* against *Altermonas* sp. and *Bacillu*s strain BY-9, similar to those reported by Abraham^65^ against *Altermonas* sp. and *Bacillus* strain BY-9 against the pathogenic V. harveyi. In another study, Galindo^66^ discovered that *L. plantarum* produced a wide range of inhibitory zones against *A. hydrophila*, while *L*. *lactis* inhibited *A. hydrophila*, *E. tarda*, and *S. aureus*. Antibiotics, antimicrobial peptides, bacteriocins, siderophores, lysozymes, and other antimicrobial peptides have all been discovered to stop pathogen growth hydrogen peroxide and organic acids, proteases^67^.

The reduction in pathogen growth and cell densities in antagonistic assays, according to Nakamura et al.^68^, was due to the bacterium's extracellular bacteriolytic product. The selection of potential probiotic strains frequently included the in vitro synthesis of chemicals that inhibit recognised diseases. G9 inhibited all five pathogenic bacteria in the current in vitro study, so it was chosen to test the strain's probiotic efficacy. The pathogenicity of G9 was determined, and there was no evidence of injury to *Mugil cephalus* in terms of mortality, disease, or abnormalities. To test the pathogenicity of the probiotic strain, Brunt and Austin^62^ used a similar immersion assay in rainbow trout for 14 days with commercial fish feed supplemented with putative probiotics. According to Austin et al.^69^, isolated probiotic strains were non-pathogenic.

The non-pathogenic characteristics of strain G9 ensures the strain's safety when used as a probiotic. Using 16S rRNA gene sequencing and a homology search, the G9 strain was biochemically identified and molecularly characterised. The BLAST database was given the name *B. subtilis* since it shared the highest identity with *B. subtilis*. *Bacillus* is a rod-shaped, strict or facultative aerobe that is catalase positive, which distinguishes it from other endospore-forming bacteria. Bacilli that are not autochthonous to the gastrointestinal system have been detected in carps^70^, coastal fishes^56,71^, bivalves^72^, shrimp culture ponds^73,74^, and shrimp larvae rearing medium^75,76^.

Difficidin, oxydifficidin, bacitracin, polymyxin, subtilin, mycobacillin, gramcidin, or bacillomycin B are antibiotics produced by Bacilli that are antibacterial in vivo and in vitro^57,58,77,78^. *Bacilli* are widely acknowledged and used as probionts, and they are unrelated to sickness in aquatic creatures^79^. In vitro situation *Bacillus subtilis* strain PRBD 09 (KF765648), isolated from the intestines of striped mullet, had significant antibacterial activity against fish illnesses As a result, this strain could be a good option for a probiotic organism in vivo, boosting the fish's immune. The strain's in vivo effectiveness will be discussed. Fish farming is becoming more popular around the world as a way to make up for the lack of animal protein. Fish in intense culture are severely harmed and frequently succumb to a variety of microbial infections that have been treated with chemotherapeutic drugs, including antibiotics. On the one hand, these curative compounds cause bacterial drug resistance, and on the other, they pose public health risks^80^.

Because of these limitations, fish pathologists begin looking for alternate options. Because biological products, such as probiotics, increase fish health and alter the microbial population associated with fish, disease bio-control tactics in aquaculture have recently been a target of biological products, such as probiotics, either alone or in combination with prebiotics^81^. The primary line of defence against invading viruses in fish is the innate immune system^82^. Innate humoral components include antimicrobial peptides, lysozyme, complement, transferrin, pentraxins, lectins, anti-proteases, and natural antibodies.

Probiotics work with a wide range of immune cells to improve immune responses. Mucus and serum protein were significantly higher in *Labeo rohita* fed a *B*. *subtilis* supplemented food, *Catla catla* fed a *B. amyloliquefaciens* supplemented diet, and *Catla catl*a fed a *B. subtilis* contained diet, according to certain studies^83,84^. To boost the growth performance and immunological response of experimental fish, probiotics have been used as nutritional supplements or water additives. *B. subtilis* was shown to efficiently protect fish against aggressive *Aeromonas* sp., making it a probiotic agent. The fact that *B. subtilis* was isolated from the gut of an apparently healthy *Mugil cephalus* supports the theory that gut microbes play a significant role in the host fish's health^85^. *B. subtilis* has shown antibiosis against pathogenic *Vibrio* sp. and has been utilised to enhance pond water quality, resulting in improved black tiger prawn survival^86^.

The growth and feed utilisation of *Mugil cephalus* fed *B. subtilis* (KF765648) supplemented diets were considerably superior than those of the fish fed the control basal diets. As a result, the probiotic *B. subtilis* appears to have growth-promoting effects (KF765648). Statistical study of numerous growth indices of *Mugil cephalus* at the end of the trial period demonstrated a significant increase in body weight gain (W.G.) in the fish fed probiotic diet as compared to the control group. The specific growth rate (SGR) in *Mugil cephalus* fed probiotic diet was also shown to be higher than in the control group, as W.G. As a result, the Condition Factor (CF) of the probiotic-supplemented fish remained higher than that of the control group. Only *Mugil cephalus* fed the basal diet (control) had a greater feed conversion ratio (FCR) than those provided probiotic supplemented diets, showing that probiotic supplemented diets are beneficial.

The highest FCR values observed in probiotic-supplemented diets indicated that probiotics increased feed consumption, which is consistent with the idea that probiotic use can reduce the amount of feed required for animal growth, thereby cutting production costs. Similar findings were observed by Lara-Flores et al.^87^. The obtained results could be linked to *B. subtilis*' ability to adhere to the intestinal mucosa of *Mugil cephalus* and produce a wide range of digestive enzymes (amylase, lipase, and protease) capable of denaturing indigestible components in the diets.

Kennedy et al.^88^ discovered that adding *B. subtilis* to the diet of common snook, *Centropomus undecimalis*, increased food absorption by boosting the protease level, resulting in greater growth. Another example is *El-haroun* et al.^89^. According to his research, *B. subtilis* germinates in the intestine of fish using a large amount of sugar (carbohydrates) and produces a wide range of digestive enzymes (amylase, lipase, and protease) that have beneficial effects such as enhanced growth rate and feed efficiency.. Similar findings were found in studies on gilthead seabream (*Sparusaurata* L.)^71,72,90,91^, tilapia (*Oreochromis niloticus*), and giant yellow croaker (*Larimichthyscrocea*)^92^.

Bacterial secretory proteins are recognised to undertake a variety of important "remote-control" tasks, including nutrition delivery, cell-to-cell communication, environmental detoxification, and competitor elimination. Pathogenic bacteria's extracellular proteins, in particular, appear to play an important role in virulence^93^. The gram-positive bacterium *B. subtilis* is a good choice for protein secretion proteomic studies since its proteins are not retained by an outer membrane. Despite the fact that proteomic approaches have uncovered a number of secretome secrets in *B. subtilis*' protein secretion mechanism^94^. These include cytoplasmic protein export, native membrane protein digestion by type I signal peptidase (SPase), and the release of cell-associated lipoproteins and cell wall proteins into the growth media. In this study, ammonium sulphate precipitation was used to begin the purification of extracellular protein from *B. subtilis* strain PRBD 9. The precipitated protein was collected, concentrated, and dialyzed.

The dialyzed proteins were purified further using G-50 (Sephadex) gel filtration chromatography, yielding a purified protein. The extracellular protein was then separated using SDS PAGE and stained with colloidal CBB. The CBB staining yielded five bands with molecular weights ranging from 29 to 66 kDa. Prior to MALDI TOF/TOF MS MS analysis, purified protein samples were subjected to in-gel trypsin digestion.

The peptide sequence for the five samples was predicted using peptide mass finger printing and Mascot search results. Hirose et al.^95^ used similar findings on extracellular proteins from *B. subtilis* to identify 101 proteins from *B. subtilis* CH97 using peptide mass finger printing. He looked at the extracellular proteome of *B. subtilis* and *B. amyloliquefaciens* in comparison.

The secretary proteome was effectively discovered using MALDI TOF/TOF analysis, resulting in the identification of five differentially expressed protein bands. Samples 08 and 09 were found to be hypothetical proteins among these proteins. Extrapolating a protein's experimentally known function from one organism to another using protein sequence similarity is the most prevalent method for attributing a function to novel genes. They are classified as hypothetical proteins because the function of one-third to one-half of the predicted proteins cannot be determined using this sequence-based gene annotation. The existence of putative protein from *B. subtilis* extracellular secretion was previously reported in the strains *B. subtilis* KB-1111 and KB-1122. Further research would be required to confirm the role of these proteins^96^.

The enzyme cytidinedeaminase, which deaminates cytidine to uridine and plays a crucial role in a range of pathways from bacteria to humans, was found in sample 010. Only in a mononucleotide or nucleoside setting were ancestors of this family able to deaminatecytidine. Recently, a class of enzymes with the ability to deaminatecytidines on RNA or DNA was found. The first member of this new family is APOBEC1, which generates a premature stop codon by deaminateing apolipoprotein B messenger RNA. This protein family has been reported to have a key role in innate antiviral immunity^97^. *Bacillus subtilis*, a probiotic bacteria, was discovered to be a dominating gut microbiota, and cytidinedeaminase from *Bacillus subtilis* has been purified 700-fold, and the role of cytidinedeaminase is considerable^98^.

The transcription repair coupling factor was discovered in sample 011. TRCF (transcription-repair coupling factor) is a big, multi-domain SF2 ATPase that has been found in a variety of bacteria. It connects nucleotide excision repair and transcription by releasing blocked RNA polymerase units from template DNA lesions. Despite the fact that eukaryotic TCR has just recently been reconstituted in vitro^99^, bacterial TCR has been extensively studied in E. coli, where the mfd gene product was identified as the sole Transcription-Repair Coupling Factor (TRCF) by in vitro complementation with purified protein^100^.

The Mfd protein binds to DNA in a sequence-independent manner and has a low ATPase activity. Furthermore, the mfd-rec strains' severe recombination-deficient phenotype implies that Mfd protein is important in homologous DNA recombinationA secreted enzyme related to transcription regulators was previously found in *B. amyloliquefaciens*^101^. Furthermore, the search identified sample 012 as Sirtuin or Sir2 proteins, which are a class of mono-ADP ribosyltransferase or deacylase proteins that include deacetylase, desuccinylase, demalonylase, demyristoylase, and depalmitoylase^102^.

Sirtuins regulate essential biological processes in bacteria, archaea, and eukaryotes. Sir2 is a cellular regulator derived from the yeast gene 'silent mating-type information regulation 2'. In low-calorie environments, sirtuins have been connected to a number of cellular activities, including ageing, transcription, apoptosis, inflammation, stress tolerance, energy efficiency, and alertness^103^. Sirtuins are also involved in the regulation of mitochondrial biogenesis and circadian clocks. Surprisingly, extracellular proteins linked to amino acids essential in protein synthesis were discovered in this study of *Bacillus* strains. This also supports the notion that the proteins in the extracellular media derived from the lysis of bacteria^104^.

The emergence of cytosolic proteins in extracellular preparations was most likely owing to cell breaking or cytoplasmic protein leaking by an unknown function, according to the current findings. These phenomena could be linked to *B. subtilis* secretome secrets, such as the apparent export of cytoplasmic proteins into the growing media^105^.

## Conclusion

The antibacterial bacterial activates of the probiotic bacterium (*B. subtilis* KF765648) isolated from the *Mugil cephalus*'s gut exhibited potential activities against a variety of fish infections in this investigation. When *B. subtilis* (KF765648) was supplemented with fish diet, it improved *Mugil cephalus* innate immune response. Fish given the probiotic *B. subtilis* (KF765648) fared better against pathogen infection, with survival rates ranging from 88 to 100%. Cytidinedeaminase is a partially discovered extracellular protein. In the future, we will concentrate our probiotic bacterial research on challenge experiments in *Mugil cephalus.*
