# Author Correction: Influence of extracellular protein isolated from fish gut associated bacteria as an enhancer of growth and innate immune system in *Mugil cephalus*

**DOI:** 10.1038/s41598-022-10147-6

**Published:** 2022-04-06

**Authors:** Rajasekar Thirunavukkarasu, Priyadarshin Pandi, Deivasigamani Balaraman, Fadwa Albalawi, Naushad Ahmad, Mani Panagal, Tentu Nageswara Rao, Kumaran Subramanian, Edward Gnana Jothi George, MaryShamya Arockia Rajan, Pugazhvendan Sampath Renuga, Wilson Aruni, Suliman Yousef AlOmar

**Affiliations:** 1 Centre for Drug Discovery and Development, Sathyabama Institute of Science and Technology, Chennai, Tamil Nadu 600 119 India; 2 Centre of Advanced Study (CAS) in Marine Biology, Annamalai University, Parangipettai, Cuddalore, Tamil Nadu 632502 India; 3 Department of Zoology, King Saud University, Riyadh, 11451 Kingdom of Saudi Arabia; 4 Chemistry Department, College of Science, King Saud University, Riyadh, 11451 Kingdom of Saudi Arabia; 5 Department of Biotechnology, Annai College of Arts & Science, Kumbakonam, Tamil Nadu India; 6 Department of Chemistry, Krishna University, Machilipatnam, Andhra Pradesh 521001 India; 7 Kemin Industries South Asia Private Limited, KeminAquaScience™, Ambattur Industrial Estate, Chennai, 600 058 India; 8 Arignar Anna Government Arts College, Cheyyar, Tamil Nadu 604407 India; 9 Amity University, Mumbai, Maharashtra India; 10 Department of Biotechnology, Mohamed Sathak College of Arts & Science, Sholinganallur, Chennai, Tamil Nadu 600119 India

Correction to: *Scientific reports* 10.1038/s41598-022-05779-7, published online 25 February 2022

In the original version of this Article Suliman Yousef AlOmar was incorrectly affiliated with ‘Chemistry Department, College of Science, King Saud University, Riyadh 11451, Kingdom of Saudi Arabia’. The correct affiliation is listed below.

Department of Zoology, King Saud University, Riyadh 11451, Kingdom of Saudi Arabia.

The original Article has been corrected.

